# Cystatin C Levels in Middle-Aged Patients with Obstructive Sleep Apnea Syndrome

**DOI:** 10.1155/2016/8081723

**Published:** 2016-10-24

**Authors:** Kostas Archontogeorgis, Evangelia Nena, Christina Tsigalou, Athanasios Voulgaris, Maria Xanthoudaki, Marios Froudarakis, Paschalis Steiropoulos

**Affiliations:** ^1^Master Program in Sleep Medicine, Medical School, Democritus University of Thrace, Alexandroupolis, Greece; ^2^Laboratory of Hygiene and Environmental Protection, Medical School, Democritus University of Thrace, Alexandroupolis, Greece; ^3^Laboratory of Biopathology, University General Hospital of Evros, Alexandroupolis, Greece; ^4^Department of Pneumonology, Medical School, Democritus University of Thrace, Alexandroupolis, Greece

## Abstract

*Background*. Obstructive sleep apnea syndrome (OSAS) is associated with systemic inflammation and increased risk of cardiovascular and chronic kidney disease. Cystatin C (Cyst C) is a novel biomarker of both latent renal damage and cardiovascular disease. Aim of the study was to measure serum levels of Cyst C, as well as IL-8 and CRP, in otherwise healthy OSAS patients.* Methods*. 84 individuals examined with polysomnography for OSAS symptoms without known comorbidities were prospectively recruited.* Results*. According to apnea hypopnea index (AHI) subjects were divided in two groups: OSAS group (AHI > 5/hour, *n* = 64) and controls (AHI < 5/hour, *n* = 20), which were age- and BMI-matched. Cyst C levels were higher in OSAS patients versus controls (1176.13 ± 351.33 versus 938.60 ± 245.83 ng/mL, resp.; *p* = 0.017) while serum IL-8 and CRP levels did not differ significantly. Positive correlation was found between Cyst C levels and respiratory disturbance index (RDI) (*r* = 0.240, *p* = 0.039) and percentage of time with oxygen saturation <90% (*r* = 0.290, *p* = 0.02) and negative correlation was found between Cyst C levels and average oxygen saturation during sleep (*r* = −0.291, *p* = 0.012). After adjustment for age and BMI, RDI was the only independent predictor of Cyst C levels (*β* = 0.256, *p* = 0.039).* Conclusion*. Cyst C serum levels are increased in OSAS patients without comorbidities, suggesting an increased renal and cardiovascular disease risk.

## 1. Background

Obstructive sleep apnea syndrome (OSAS) is characterized by recurrent episodes of upper airway collapse, resulting in oxygen desaturation and sleep fragmentation [1]. It is a highly prevalent disorder affecting approximately 10–17% of men and 3–9% of women; however it often remains undiagnosed mainly due to lack of awareness and limited access to sleep laboratories [2]. OSAS is associated with increased cardiovascular and cerebrovascular morbidity [3, 4]. Oxygen desaturation, caused by apneic events, together with arousals, negative intrathoracic pressure, and repeated activation of the sympathetic system, activates a series of neural, humoral, thrombotic, and metabolic responses that may trigger atherosclerosis [5, 6].

OSAS has been also associated with chronic kidney disease (CKD) [7]. There is increased prevalence of OSAS in patients with CKD, ranging between 41% and 65% in various studies [8, 9]. On the other hand, CKD is more common among OSAS patients, with prevalence estimated at 18% for patients with severe OSAS [10]. Pathogenetic mechanisms that contribute to the development of the syndrome in this population setting may include fluid overload and rostral displacement of fluid [11], aldosterone excess [12], hyperactivation of the sympathetic nervous system [12], and destabilization of respiratory control [13]. Furthermore, OSAS may play an indirect role in the development and progression of CKD by sharing and exacerbating common risk factors such as arterial hypertension, diabetes mellitus, and obesity [14].

Kidney function impairment has been studied in OSAS and low grade albuminuria was considered as a marker for subclinical vascular damage in these patients [15]. Cystatin C (Cyst C) is a cysteine proteinase inhibitor that is produced by all nucleated cells, is freely filtered by the glomerulus, and is reabsorbed and catabolized in the proximal tubule, but it is not secreted by the tubules [16, 17]. Thus, serum Cyst C concentration is thought to depend almost completely on the glomerular filtration rate (GFR). Data from previous research suggests that serum Cyst C is superior to serum creatinine as a marker of kidney function when GFR is used as a reference standard [18]. Additionally, Cyst C has emerged as a potential indicator of cardiovascular risk [19]. In OSAS patients without CKD, serum Cyst C was found increased, reflecting latent renal dysfunction as well as augmented cardiovascular risk [20].

There is increasing evidence that inflammatory processes, triggered by intermittent hypoxia and reoxygenation, play an important role in the development of cardiovascular disease in OSAS [21]. In addition, a variety of serum inflammatory markers were found increased in untreated patients with OSAS, while their levels decreased after continuous positive airway pressure treatment [22, 23]. C-reactive protein (CRP) is an inflammatory marker produced by the liver in response to interleukin-6 and its serum levels increase as a consequence of trauma or infection. Considerable evidence suggests an independent association between serum CRP levels and OSAS [24]. Interleukin-8 (IL-8) is a chemokine produced mainly by macrophages and other cells, such as epithelial cells, airway smooth muscle cells, and endothelial cells [25]. Previous studies reported high serum IL-8 levels in patients with OSAS that decreased after treatment with CPAP [26].

The purpose of this study was to investigate the possible risk of latent renal function impairment and cardiovascular disease in otherwise healthy OSAS patients. To this purpose we evaluated serum Cyst C, interleukin-8 and CRP levels in newly diagnosed OSAS patients without other known comorbidities.

## 2. Methods

### 2.1. Patients

Included were patients referred to the sleep laboratory of our institution with symptoms suggesting sleep-related breathing disorders. Detailed data regarding previous medical history, current medication use, and tobacco smoking were obtained. A clinical examination was performed and anthropometric characteristics were measured. Height, weight, neck circumference, hip, and waist circumference and waist/hip circumference ratio were measured using a standardized protocol. Body mass index (BMI) was calculated using the following formula: BMI = weight (kg)/height^2^ (m). Blood pressure was recorded as the average of three consecutive measurements in the seated position. Sleepiness was evaluated using the Greek version of the Epworth Sleepiness Scale (ESS) [27], a self-administered questionnaire evaluating the possibility of falling asleep in a variety of situations [maximum score: 24; score >10: excessive daytime sleepiness]. Pulmonary function testing, arterial blood gases analysis, and a 12-lead electrocardiogram were also performed for the exclusion of pulmonary and cardiovascular disease. Patients with the following characteristics were excluded from the study: patients with exclusively central sleep apneas in the polysomnography, hypertensive patients (defined as systolic blood pressure ≥140 mmHg, diastolic blood pressure ≥90 mmHg, or previously diagnosed/under treatment), patients with previously diagnosed diabetes mellitus, patients with known OSAS under treatment with CPAP or oral appliances, and patients with known kidney disease. All subjects provided their consent, after being informed about the goals and the procedures of the study. The study protocol was approved by the institutional ethics committee.

### 2.2. Polysomnography

Overnight polysomnography (PSG), attended by an experienced sleep technician, was performed from 22:00 to 06:00 hours and variables were recorded on a computer system (Alice® 4, Philips Respironics, Murrysville, PA, USA).

Apneas, hypopneas, and electroencephalogram recordings were manually scored according to standard criteria [28]. Apnea was defined as a complete cessation of airflow for at least 10 sec [28]. Hypopnea was defined as a 50% reduction in airflow for at least 10 sec in combination with oxyhaemoglobin desaturation of at least 4% or an arousal registered by the electroencephalogram. The average number of apneas and hypopneas per hour of PSG-recorded sleep time was calculated as the apnea-hypopnea index (AHI) [28]. The respiratory disturbance index (RDI) is defined as the average number of respiratory disturbances (apneas, hypopneas, and respiratory event-related arousals (RERAs)) per hour of PSG-recorded sleep time [28]. Subjects with AHI <5/h of sleep were considered as controls. OSAS was defined as AHI ≥5/h accompanied by symptoms [1]. OSAS was graded as mild (AHI: 5–15/h), moderate (AHI: 15–30/h), and severe (AHI > 30/h) [1].

### 2.3. Blood Sampling and Laboratory Analysis

Venous blood samples were collected the morning after PSG from the antecubital vein after at least 8 hours of overnight fasting, were immediately centrifuged (10 minutes at 3000 rpm), and were cryopreserved at −80°C until analysis. Fasting blood glucose, triglycerides, total cholesterol, high and low density lipoprotein, urea, creatinine, and CRP were calculated by a random-access chemistry analyser (AU640; Olympus; Hamburg, Germany). IL-8 and Cyst C serum concentrations were both measured by ELISA test using commercially available kits (Bender MedSystems GmbH, Vienna, Austria, for IL-8 and Biovendor, Czech Republic, for Cyst C) according to manufacturer's specifications. GFR was calculated using the abbreviated four-variable version of the modification of diet in renal disease (MDRD) formula [29, 30].

### 2.4. Statistical Analysis

All analyses were performed using version 17.0 of the IBM Statistical Package for Social Sciences (SPSS Inc. Released 2008. SPSS Statistics for Windows, Version 17.0. Chicago: SPSS Inc.). Continuous variables were tested for normality of distribution by the Kolmogorov-Smirnov test. Quantitative data with normal distribution are expressed as mean ± standard deviation (SD). Correlations were analysed with Pearson's correlation coefficient, while comparisons between means were explored with the Student's* t*-test. Logistic regression analysis with Cys C as the dependent variable was performed. Possible predictors of serum Cys C levels (such as age and BMI) were entered into the regression and then polysomnographic parameters were added to the model. Two-tailed significance was defined at *p* < 0.05 level.

## 3. Results

A total of 84 subjects (68 men and 16 women) participated in the study. Participants were divided according to their AHI in two groups: OSAS group (AHI > 5/h) that included 64 patients (52 men and 12 women) and control group (AHI < 5/h) that included 20 individuals (16 men and 4 women). Groups did not differ in terms of age (51.78 ± 11.55 for OSAS patients versus 51.40 ± 16.24 years for control group, *p* = 0.944) and BMI (36.34 ± 13.18 for OSAS patients versus 33.73 ± 5.68 kg/m^2^ for control group, *p* = 0.308). Anthropometric characteristics of the two groups are presented in Table 1, while sleep characteristics are presented in Table 2.

No significant differences between the two groups were revealed, regarding pulmonary function, arterial pressure, blood urea, creatinine, glucose, lipidemic profile, and eGFR. Serum Cys C levels were higher in OSAS patients compared with controls (1176.13 ± 351.33 versus 938.60 ± 245.83 ng/mL, resp., *p* = 0.017), while serum IL-8 levels and CRP levels did no differ between groups (31.79 ± 20.09 versus 34.09 ± 14.37 pg/mL, *p* = 0.665 and 0.55 ± 0.57 versus 0.32 ± 0.41 mg/dL, *p* = 0.154, resp.). Results of all laboratory analyses performed are presented in Table 3.

**Figure 1:**
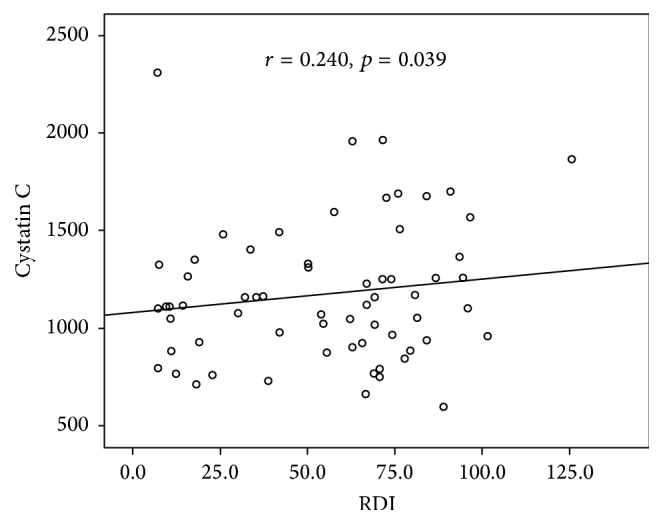
Correlation between Cystatin C levels and respiratory disturbance index (RDI) in OSAS patients.

**Figure 2:**
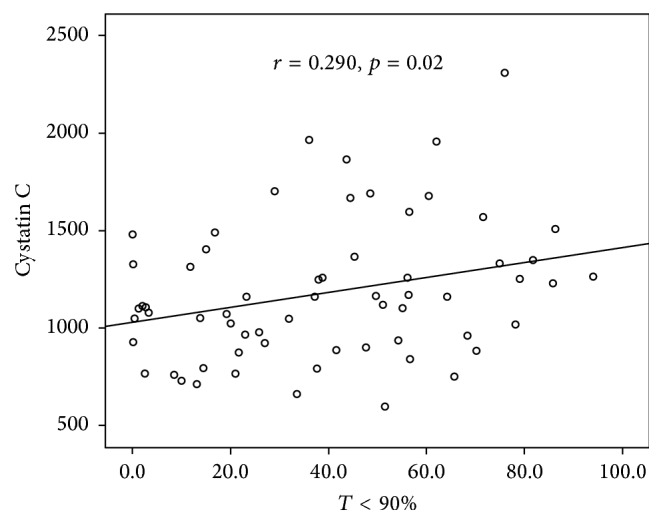
Correlation between Cystatin C levels and percentage of time with oxyhaemoglobin saturation <90% (*T* < 90%) during sleep in OSAS patients.

**Figure 3:**
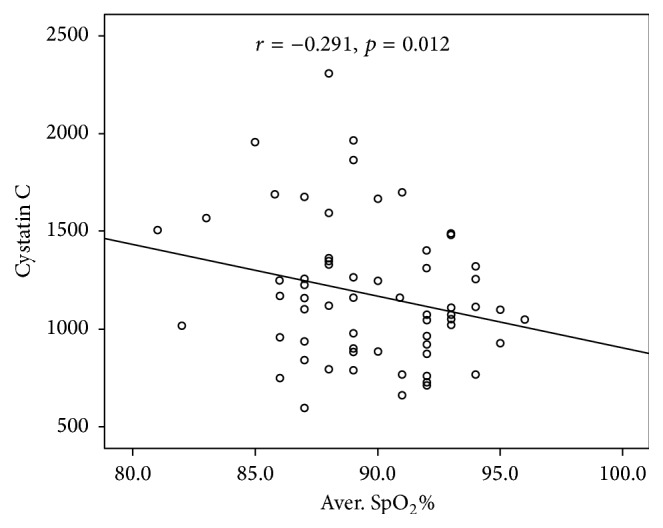
Correlation between Cystatin C levels and average oxyhaemoglobin saturation during sleep in OSAS patients.

In the OSAS group, no significant correlation between serum Cys C levels and anthropometric parameters was observed. Serum Cys C levels were positively correlated with RDI (*r* = 0.240, *p* = 0.039) (Figure 1) and percentage of time with oxyhaemoglobin saturation <90% during sleep (*r* = 0.290, *p* = 0.02) (Figure 2) and negatively correlated with average oxyhaemoglobin saturation (*r* = −0.291, *p* = 0.012) (Figure 3). Correlation analysis results between serum Cys C levels and anthropometric and sleep parameters are presented in Table 4. No correlation was found between serum Cys C levels and CRP (*r* = 0.174, *p* = 0.114) or IL-8 levels (*r* = 0.184, *p* = 0.114).

Serum CRP levels were not correlated with anthropometric or sleep characteristics of OSAS patients. Likewise, no correlation was observed between serum IL-8 levels and sleep parameters. A significant negative correlation was revealed between IL-8 levels and age. After adjustment for age and BMI, a significant correlation was revealed between RDI and serum Cys C levels (*β* = 0.256, *p* = 0.039) (Table 5).

## 4. Discussion

The present study is the first one showing that serum Cyst C levels are elevated in middle-aged OSAS patients without known comorbidities, in comparison to age- and BMI-matched healthy controls. Furthermore, serum Cyst C levels were correlated with RDI and indices of hypoxia such as time with oxyhaemoglobin saturation <90% and average oxyhaemoglobin saturation during sleep. These results suggest a probable increased risk for renal and cardiovascular disease in OSAS patients and nocturnal hypoxia appears to be implicated in the pathogenetic mechanism.

Cyst C is a novel biomarker with greater sensitivity, compared to serum creatinine, for the detection of latent renal damage [31]. This property mostly depends on its constant production, which remains unaffected from muscle mass, age, sex, and the absence of renal secretion or resorption [31]. Cyst C serum levels are also associated with increased risk for cardiovascular disease. In a large study that evaluated the role of early kidney dysfunction as a risk factor for hypertension that included 2,767 individuals with a median follow-up of 3.1 years, higher Cyst C levels were associated with older age and traditional cardiovascular risk factors [32]. In the same study, after adjustment for established arterial hypertension risk factors, each increase in Cyst C serum levels of 15 nmol/L was associated with a 15% greater incidence of hypertension (*p* = 0.017) [32]. A meta-analysis that included 9 studies showed that elevated serum Cyst C levels were independently associated with excessive cardiovascular mortality (HR 2.74, 95% CI 2.04–3.68) and each standard deviation increment augmented 57% cardiovascular mortality risk (HR 1.57, 95% CI 1.31–1.88) [33].

Previous studies examined the association between OSAS and serum Cyst C levels. In the study of Kato et al. [20] that included 267 patients, Cyst C levels were correlated with age (*r* = 0.37, *p* < 0.001), BMI (*r* = 0.12, *p* = 0.045), AHI (*r* = 0.17, *p* = 0.007), CRP (*r* = 0.12, *p* = 0.045), and brachial-ankle pulse wave velocity (*r* = 0.18, *p* = 0.003), while severe OSAS was an independent variable for the highest quartiles of serum Cyst C levels (OR: 2.04, 95% CI: 1.04–4.01, *p* = 0.04) after adjustment for age, BMI, hypertension, and diabetes mellitus. Similarly, in another study that included hypertensive patients with OSAS, age (OR = 1.996, 95% CI = 1.366–2.917), blood pressure control (OR = 2.895, 95% CI = 1.267–6.615), and severe OSAS (OR = 6.093, 95% CI = 1.267–29.303) were the influencing factors for Cyst C plasma levels [34]. In the study of Zhang et al. [35], 3 months of CPAP treatment significantly reduced Cyst C serum levels in patients with severe OSAS (0.87 ± 0.18 versus 0.77 ± 0.21 mg/L, *p* = 0.000), but creatinine levels and eGFR were not affected. In another study that included fifty patients with chronic heart failure and sleep disordered breathing, adaptive servoventilation (ASV) significantly improved AHI, central apnea index, obstructive apnea index, arousal index, and mean and lowest hemoglobin saturation compared to baseline and reduced NT-proBNP and Cyst C plasma levels (1.391 ± 0.550 at baseline versus 1.348 ± 0.489 mg/L after ASV, *p* < 0.05 for Cyst C) [36]. However, populations enrolled in all these studies included patients with comorbidities, such as hypertension and diabetes mellitus, both conditions associated with increased Cyst C serum levels [19, 32].

Zhang et al. [37] studied the association between serum Cyst C levels and OSAS in younger patients (age ≤40 years) without comorbidities. To this purpose, 98 subjects were recruited (mean age 32.5 years) and were divided according to their AHI in mild, moderate, and severe OSAS and control groups. Patients with severe OSAS had higher serum Cyst C levels compared to controls (0.87 ± 0.12 versus 0.74 ± 0.1 mg/L, resp., *p* < 0.05). Cyst C correlated with AHI (*r* = 0.319, *p* = 0.001), oxygen desaturation index (ODI) (*r* = 0.279, *p* = 0.005), high sensitivity CRP (*r* = 0.321, *p* = 0.001), serum creatinine (*r* = 0.233, *p* = 0.021), and eGFR (*r* = −0.241, *p* = 0.017). After adjustment for confounding factors, AHI was positively associated with serum Cyst C levels (*β* = 0.284, *p* = 0.007). However, severe OSAS patients had significantly higher BMI compared to the control group (29.69 ± 3.81 versus 26.42 ± 3.10, *p* < 0.05). On the other hand, the lack of difference in terms of BMI between OSAS patients and controls in our study population eliminated obesity as a confounding factor. In addition to this, mean age of 51.8 years in our study group is more representative for OSAS.

Serum IL-8 levels did not differ between OSAS patients and controls. IL-8 is a chemokine linked with inflammatory processes and the pathogenesis of coronary disease and atherosclerosis [38]. Its production is mainly controlled by other soluble factors such as interleukin-1 and tumor necrosis factor-*α* and is stimulated by hypoxia [39, 40]. The association between IL-8 blood levels and OSAS remains ambiguous. In a study that included 25 patients with severe OSAS and 17 healthy individuals of similar age and BMI, IL-8 serum levels were increased in OSAS patients compared with controls (198.8 ± 4.76 versus 180.83 ± 3.38 pg/mL, resp., *p* < 0.005) [41]. Similarly, in the study of Ohga et al. [26] IL-8 circulating levels were increased in OSAS patients compared with healthy controls (*p* < 0.05) and were correlated with AHI (*r* = 0.51, *p* = 0.004) and desaturation magnitude (*r* = 0.69, *p* < 0.001). Moreover, CPAP treatment for 8 months significantly decreased serum IL-8 levels in OSAS patients (*p* < 0.05 versus baseline). Our findings are in agreement with previous reports which failed to show a connection between OSAS and IL-8 levels. In the study of Kim et al. [42] IL-8 serum levels were similar between OSAS patients and healthy controls (*p* = 0.38). In another comparative study, IL-8 produced by peripheral blood mononuclear cells as well as IL-8 circulating levels did not significantly differ between 16 OSAS patients and 11 healthy subjects (*p* = 0.43). In the same study, 12 weeks of CPAP therapy did not alter chemokine levels (*p* = 0.98) [43].

In the present study, there was no association between CRP serum levels and OSAS. Several investigators studied the relationship between CRP and OSAS presenting conflicting results [22]. Some studies reported increased CRP serum levels in OSAS patients, but other studies failed to verify this association and suggested that external factors, such as obesity, may influence the results. Yokoe et al. [44] had found increased CRP serum levels in 30 OSAS patients compared with 14 obese nonapneic subjects (0.21 ± 0.02 versus 0.07 ± 0.01 mg/dL, *p* < 0.0001). However, in this study OSAS patients had higher BMI versus non-OSAS subjects and the control group included patients with comorbidities such as arterial hypertension, diabetes, and cardiovascular disease, all conditions influencing CRP levels. In the study of Sharma et al. [45] that included 97 subjects, hs-CRP was correlated with BMI (*r* = 0.25, *p* = 0.01) but not with AHI (*r* = 0.16, *p* = 0.12). After adjustment for BMI and age, hs-CRP levels did not correlate significantly with AHI (*r* = 0.10, *p* = 0.33). Similarly, a large study including 907 OSAS patients failed to demonstrate an association between CRP and AHI after adjustment for BMI (2.50 [95% CI: 2.12–2.92], *p* = 0.32 for AHI 5–15 and 2.61 [95% CI: 2.17–3.10], *p* = 0.76 for AHI ≥ 15/h) [46]. Although our study included OSAS patients without comorbidities, we did not observe an association between CRP levels and disease severity.

The results of the present study imply an increased risk of kidney disease in OSAS patients. Intermittent hypoxia, a key feature in OSAS, seems to be implicated in the pathological mechanism as Cyst C levels were positively correlated with time with oxyhaemoglobin saturation <90% and negatively correlated with average oxyhaemoglobin saturation during sleep.

The effect of nocturnal hypoxia in kidney disease was studied in 858 subjects referred for diagnostic testing of sleep apnea who had serial measurement of their kidney function. Nocturnal hypoxia was defined as oxygen saturation below 90% for more than 12% of the nocturnal monitoring time. In this patient cohort, 374 (44%) had nocturnal hypoxia and 49 (5.7%) had accelerated loss of kidney function. When compared to controls without hypoxia, patients with nocturnal hypoxia presented with an increased risk for accelerated kidney function loss (reduction in eGFR by ≥4 mL/min/1.73 m^2^ per year) with an odds ratio of 2.89 (95% CI: 1.25–6.67) after adjustment for confounding factors [47]. Additional proposed mechanisms include sympathetic hyperactivity, glomerular dysfunction due to arterial hypertension, and endothelial damage caused by oxidative stress and inflammation [48]. Moreover, both OSAS and kidney disease share common risk factors such as obesity, diabetes, and arterial hypertension.

Our study has indeed certain limitations. First, it has a cross-sectional design; thus long term follow-up is required in order to define the prevalence of renal damage in OSAS patients with increased serum Cyst C levels. Secondly, the accuracy of estimated GFR is limited because serum creatinine concentration is affected by factors external to creatinine filtration [49]. However, direct measurement of GFR is difficult to perform in everyday clinical practice. Finally, we used measurements of CRP and not the most accurate high sensitivity CRP (hs-CRP) levels.

In summary, in middle-aged OSAS patients without known comorbidities higher levels of serum Cyst C were revealed compared with nonapneic age- and BMI-matched controls, indicating a probable higher risk of developing chronic kidney and cardiovascular disease in this group. Intermittent hypoxia seems to play a central role in the progression of this process. Further research is necessary to better define the significance of the interaction between serum Cyst C levels and kidney disease in OSAS patients.

## Figures and Tables

**Table 1:** Comparison of anthropometric characteristics between OSAS patients and controls.

	OSAS patients (AHI > 5/h) *n* = 64	Controls (AHI < 5/h) *n* = 20	*p*
Gender (male/female)	52/12	16/4	0.680
Age (years)	51.78 ± 11.55	51.40 ± 16.24	0.944
BMI (kg/m^2^)	36.34 ± 13.18	33.73 ± 5.68	0.308
Neck circumference (cm)	44.18 ± 4.15	39.66 ± 4.84	0.072
Waist circumference (cm)	121.58 ± 19.72	110.91 ± 22.30	0.308
Hip circumference (cm)	120.85 ± 14.73	115.16 ± 16.50	0.453
Waist to hip ratio	1.02 ± 0.69	0.96 ± 0.06	0.106
Smoking (%)	33	31	0.538

BMI: body mass index and OSAS: obstructive sleep apnea syndrome.

**Table 2:** Comparison of sleep characteristics between OSAS patients and controls.

	OSAS patients (AHI > 5/h) *n* = 64	Control group (AHI < 5/h) *n* = 20	*p*
Recording time (min)	366.95 ± 62.54	381.40 ± 20.68	0.121
TST (min)	310.28 ± 69.40	291.95 ± 55.46	0.366
N1 (%)	20.50 ± 19.44	23.03 ± 14.97	0.643
N2 (%)	65.60 ± 14.50	53.93 ± 9.05	**0.003**
N3 (%)	6.44 ± 7.26	11.14 ± 8.67	0.133
REM (%)	8.91 ± 6.77	11.91 ± 10.19	0.390
RDI	54.83 ± 29.91	2.54 ± 1.11	**<0.001**
Aver. SpO_2_ (%)	89.69 ± 3.23	93.8 ± 2.34	**<0.001**
Min SpO_2_ (%)	69.06 ± 11.40	88.50 ± 3.74	**<0.001**
*T* < 90% (%)	38.42 ± 26.65	0.75 ± 1.53	**<0.001**
Arousal index	40.62 ± 21.49	14.34 ± 10.60	**<0.001**
Sleep efficiency (%)	81.80 ± 15.05	76.55 ± 13.87	0.293
ESS score	11.05 ± 5.54	9.70 ± 4.11	0.378

AHI: apnoea-hypopnoea index, Aver. SpO_2_: average oxyhaemoglobin saturation during sleep, ESS: Epworth sleepiness scale, Min SpO_2_: minimum oxyhaemoglobin saturation during sleep, N1: sleep stage 1, N2: sleep stage 2, N3: sleep stage 3, OSAS: obstructive sleep apnea syndrome, RDI: respiratory disturbance index, REM: rapid eye movement, TST: total sleep time, and *T* < 90%: time with oxyhaemoglobin saturation <90%.

**Table 3:** Comparison of the laboratory results between OSAS patients and controls.

	OSAS patients (AHI > 5/h) *n* = 64	Control group (AHI < 5/h) *n* = 20	*p*
FEV_1_ (% pred)	90.18 ± 21.28	99.50 ± 23.08	0.280
FVC (% pred)	92.83 ± 50.44	102.17 ± 19.32	0.315
FEV_1_/FVC (%)	82.39 ± 10.84	78.84 ± 9.40	0.322
pO_2_ (mmHg)	76.70 ± 13.24	83.87 ± 14.86	0.179
pCO_2_ (mmHg)	40.96 ± 5.44	39.41 ± 5.57	0.429
SBP (mmHg)	125.70 ± 11.10	119.17 ± 12.81	0.279
DBP (mmHg)	78.26 ± 7.70	75.00 ± 8.36	0.401
Glucose (mg/dL) (normal range: 70–105)	112.17 ± 40.72	101.50 ± 22.11	0.231
Urea (mg/dL) (normal range: 17–43)	36.50 ± 16.16	42.20 ± 23.26	0.472
Creatinine (mg/dL) (normal range: 0.8–1.4)	0.89 ± 0.15	1.00 ± 0.38	0.414
eGFR (mL/min/1.73^2^)	93.78 ± 14.85	88.78 ± 26.22	0.569
Cholesterol (mg/dL) (normal values: <200)	206.34 ± 43.28	209.70 ± 45.29	0.830
Triglycerides (mg/dL) (normal range: 40–160)	185.47 ± 110.08	177.30 ± 119.79	0.843
LDL-C (mg/dL) (normal values: <140)	121.24 ± 37.32	125.24 ± 40.25	0.774
HDL-C (mg/dL) (normal values: ≥35)	46.60 ± 12.81	48.80 ± 11.07	0.579
CRP (mg/dL) (normal values: <0.50)	0.55 ± 0.57	0.32 ± 0.41	0.154
IL-8 (pg/mL)	31.79 ± 20.09	34.09 ± 14.37	0.665
Cystatin C (ng/mL)	1176.13 ± 351.33	938.60 ± 245.83	**0.017**

CRP: C-reactive protein, DBP: diastolic blood pressure, eGFR: estimated glomerular filtration rate, FEV_1_: forced expiratory volume in 1st second, FVC: forced vital capacity, HDL-C: high density lipoprotein cholesterol, IL-8: interleukin 8, LDL-C: low density lipoprotein cholesterol, OSAS: obstructive sleep apnea syndrome, pCO_2_: partial pressure of CO_2_, pO_2_: partial pressure of O_2_, and SBP: systolic blood pressure.

**Table 4:** Correlation analysis between serum cystatin C levels and anthropometric and sleep parameters in OSAS patients.

	Cystatin C	
	*r*	*p*
BMI	−0.034	0.777
Age	0.064	0.585
Neck circumference	0.243	0.095
Waist circumference	0.248	0.089
Hip circumference	0.195	0.184
Waist to hip ratio	0.145	0.330
eGFR	−0.170	0.149
Creatinine	0.208	0.076
RDI	0.240	**0.039**
Aver. SpO_2_	−0.291	**0.012**
Min SpO_2_	−0.221	0.059
*T* < 90%	0.290	**0.02**

Aver. SpO_2_: average oxyhaemoglobin saturation during sleep, BMI: body mass index, eGFR: estimated glomerular filtration rate, Min SpO_2_: minimum oxyhaemoglobin saturation during sleep, OSAS: obstructive sleep apnea syndrome, RDI: respiratory disturbance index, and *T* < 90%: time with oxyhaemoglobin saturation <90% during sleep.

**Table 5:** Multiple regression analysis results in a model examining the predictive role of age, BMI, and RDI on serum cystatin C levels in OSAS patients.

	Cystatin C		
	*β*	*p*	*B*
Age	0.127	0.282	−2.470
BMI	−0.090	0.461	3.640
RDI	0.256	**0.039**	2.660
*R* ^2^	0.071		

BMI: body mass index, OSAS: obstructive sleep apnea syndrome, and RDI: respiratory disturbance index.
